# Filamentous Bacteria and Stalked Ciliates for the Stable Structure of Aerobic Granular Sludge Treating Wastewater

**DOI:** 10.3390/ijerph192315747

**Published:** 2022-11-26

**Authors:** Yifan Liang, Zengrui Pan, Tao Guo, Hongbo Feng, Anqi Yan, Yongjiong Ni, Jun Li

**Affiliations:** Key Laboratory of Microbial Technology for Industrial Pollution Control of Zhejiang Province, College of Environment, Zhejiang University of Technology, Hangzhou 310014, China

**Keywords:** aerobic granular sludge, filamentous bacteria, stalked ciliates, Epistylis, wastewater treatment

## Abstract

Aerobic granular sludge (AGS) is a promising technology for wastewater treatment. AGS formation belongs to microbial self-aggregation. Investigation of the formation and stability of AGS is widely paid attention to, in particular the structure stability of large size granules. Two types of AGS were developed in two sequencing batch reactors fed by two different wastewaters, respectively. Through confocal laser scanning microscope (CLSM) and scanning electron microscopy (SEM), the structure and composition of granules were analyzed. Filamentous bacteria were observed in granules from synthetic wastewater reactor, while filamentous bacteria and stalked ciliates (*Epistylis* sp.) were simultaneously found in granules from domestic wastewater reactor. The analytic results show that filamentous bacteria and stalked ciliates acting as skeletons play important roles in the formation and stability of granules. With the bonding of extracellular polymeric substances (EPS), the filamentous bacteria and stalked ciliates could build bridges and frames to promote the aggregation of bacteria; these microorganisms could create a space grid structure around the surface layer of granules to enhance the strength of granules, and the remnants of the stalks could serve as supports to fix the steadiness of granules.

## 1. Introduction

The biological method is a commonly used wastewater treatment technology [1]. Compared with physical adsorption and chemical wastewater treatment technology, it has the advantages of high efficiency and low cost [1,2]. Aerobic granular sludge (AGS) is widely considered as a special biological particles through microbial self-aggregation [3,4]. Compared to the conventional activated sludge process, AGS presents some advantages, such as higher settling velocity, better solid–liquid separation, higher biomass concentration, longer sludge retention time and higher loading rates [5,6,7]. These advantages indicate that AGS technology has great potential for various wastewater treatments [8,9,10].

The composition of AGS is mainly bacteria and EPS [11]. Aerobic granules, dwelling vorticella and rotifers were found in a sequencing batch reactor (SBR) fed with domestic wastewater [12,13]. The analytical result shows that most vorticella are anchored into the granules by stalks, while rotifers attached or adhered to the surface of granules. Flower-like vorticella on the surface of granules lead to the lower settling velocity. Nevertheless, a positive effect on the reduction of suspended solids (SS) can be linked to the ingested fine biomass particles by vorticella and rotifers. More Epistylis are attached and rooted to the granule’s cores in an aerobic granular SBR-treating domestic wastewater than in synthetic wastewater. In addition, it was reported that filamentous bacteria and protozoa act as building blocks and are beneficial to aerobic granulation [14,15].

A model was proposed for aerobic granulation as the following steps [16,17]. Firstly, there is physical movement to initiate bacterium-to-bacterium contact. Secondly, stabilization of the multicell contacts results from the initial attractive forces including physical, chemical and bio-chemical forces. Particularly, filamentous bacteria can bridge individual cells. Thirdly, the maturation of cell aggregation through production of the extracellular polymer, growth of cellular clusters, metabolic change and the environment-induced genetic effects that facilitate the cell–cell interaction result in a highly organized microbial structure. Fourthly, the shaping of the steady-state three-dimensional structure of microbial aggregate by hydrodynamic shear forces occurs. Granules can be described as a special case of biofilm formation [18].

Many strategies have been revealed for aerobic sludge granulation. The short settling time exerts a selection pressure to retain sludge with good settling ability and wash-out of light flocculent biomass [19,20]. In addition, control of organic loading rates and the operation of feast-famine in substrates could be favorable for granulation [21,22]. Furthermore, extracellular polymeric substances (EPS) secreted by microorganisms had a significant impact on formation of granules through aerobic starvation and high shear force [23,24]. Moreover, the addition of Ca^2+^ and Mg^2+^, micro-powder or aggregates as “nuclei” in sludge could also enhance granulation [25,26].

The structure of AGS at a microscopic level, the simultaneous existence of aerobic, anoxic and anaerobic conditions could be formed due to the microbial population stratification. Removal of organic matter, nitrogen and phosphorus could be achieved in a single unit with high efficiencies [27]. With the development of granules, the presence of anaerobic bacteria is likely to result in the production of organic acids and gases within the granules. These end products of anaerobic metabolisms can destroy the granules, or at least diminish their long-term stability [17]. However, the instability of AGS during long-term operation is still seen as a major barrier for a broad practical application of this technology [28].

A stable structure often depends on reliable skeletons. In our previous works, we found that some microorganisms, such as filamentous bacteria and stalked ciliates growing up in shape of filaments or stalks, could act as skeletons to support the formation and stability of AGS [12,14,29,30]. In this study, we intended to observe more detailed structures and compositions of AGS by using a confocal laser scanning microscope (CLSM) and scanning electron microscopy (SEM), to obtain a better understanding of filamentous bacteria and stalked ciliates as skeletons of AGS.

## 2. Materials and Methods 

### 2.1. Sampling

Two types of aerobic granules from SBRs were used as samples for analysis. The one from SBR1 was analyzed for the structure of filamentous bacteria in the granules and the other, from SBR2, was analyzed for the structure of protozoa in the granules.

The SBR1 fed by synthetic wastewater had a working volume of 4.2 L and a diameter of 9 cm. The influent entered through the top of the reactor, and the effluent was drawn at a volume of 2 L. The reactor’s operation was the following: influent (30 min), aeration (150 min), settling (1 min), discharge (6 min) and idle (53 min) for one cycle. The reactor operated six cycles per day. The seed sludge was taken from a municipal wastewater treatment plant. Synthetic wastewater with 850–1100 mg/L COD was supplied through the influent. The synthetic wastewater was composed as follows: CH_3_COONa (1500 mg/L), NH_4_Cl (400 mg/L), K_2_HPO_4_·3H_2_O (21 mg/L), FeSO_4_·7H_2_O (10 mg/L), MgSO_4_·7H_2_O (12 mg/L), CaCl_2_·2H_2_O (14 mg/L) and trace solution (1 mL/L). The compositions of the trace solution were: H_3_BO_3_ (100 mg/L), CoCl_2_·6H_2_O (100 mg/L), CuSO_4_·5H_2_O (30 mg/L), FeCl_3_·6H_2_O (1000 mg/L), MnCl_2_·2H_2_O (110 mg/L), ZnSO_4_·7H_2_O (100 mg/L), Na_2_Mo_7_O_24_·2H_2_O (70 mg/L), KI (30 mg/L), NiCl_2_ (60 mg/L). The upflow air velocity was 1.3 cm/sec, and more detailed experimental parameters can be obtained from our previous study [31].

The SBR2 fed by domestic wastewater had a working volume of 11 L and a diameter of 20 cm. The influent entered through the top of the reactor, and the effluent was drawn at a volume of 7 L. The reactor’s operation was the following: (10 min), aeration (120 min), settling (1 min), discharge (15 min) and idle (34 min) for one cycle. The reactor operated eight cycles per day. The seed sludge was taken from a municipal wastewater treatment plant. The raw wastewater was taken from a septic tank in a community. Due to the carbon to nitrogen (C/N) ratio of raw wastewater being too low, approximately 90 g of completely dissolved soluble starch and 60 L raw wastewater were added into a container, and then diluted to a total volume of 500 L for the influent. Moreover, approximately 10 g of potassium dihydrogen phosphate was added to maintain the PO_4_^3+^ concentration each time. The wastewater with COD (250–450 mg/L), NH_4_^+^-N (20–30 mg/L) and TP (6–11 mg/L) was supplied. The upflow air velocity was 1.2 cm/sec, and more detailed experimental parameters can be obtained from our previous study [12].

### 2.2. Analytic Methods

CLSM images were recorded using a TCS SP confocal laser scanning microscope (Leica, Germany). Granules’ bacteria were stained using the SYTO 60 nucleic acid stain (Molecular Probes, Eugene, OR, USA). The stock solution of SYTO 60 as supplied was used at a dilution of 1:1000 in deionized water. Glycoconjugates of granules were stained with the Aleuria aurantia lectin (Vector, Burlingame, CA, USA) conjugated to Alexa488 (Molecular Probes) according to a screening of all commercially available lectins on the same type of biofilms [32]. Detailed information on the methods can be found elsewhere [33].

SEM were fixed with 2.5% glutaraldehyde in 0.1 M phosphate buffer (pH = 7.3) for up to 12 h at 4 °C and post-fixed in 1% OsO_4_ at room temperature for 90 min. Further SEM sample preparations and microscopic analysis were performed in our previous studies [12,31].

Granule size, distribution of diameters, length and width of filamentous bacteria and roundness were analyzed using a Leica Digital optical microscope and Image-Pro Plus.

## 3. Results

### 3.1. Granules from SBR1 (Synthetic Wastewater)

#### 3.1.1. Morphology

**Figure 1:**
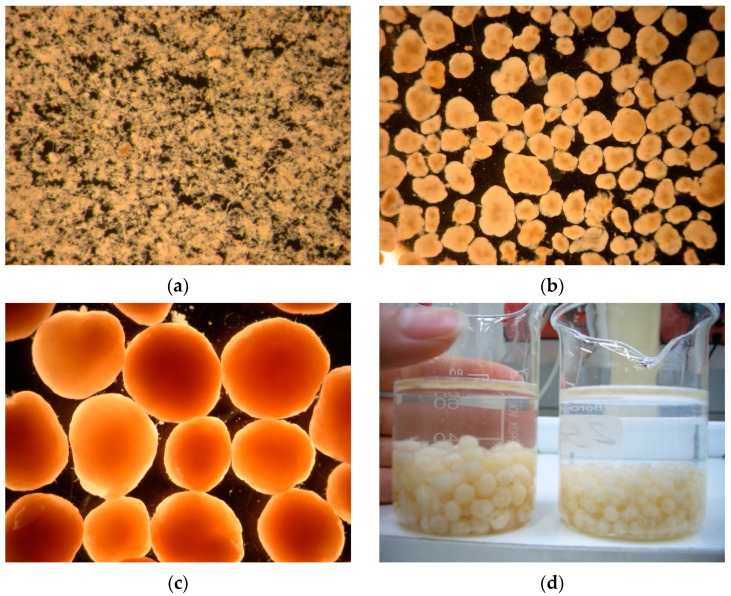
Images for development of granules with time from SBR1 (synthetic wastewater). (**a**) Day 0 (inoculum, floc sludge); (**b**) day 60; (**c**) day 150; (**d**) day 230. Scale bar = 2 mm.

The microscopic examination shown in Figure 1 presents the development of granules in morphology. The inoculum was floc sludge from a local municipal wastewater treatment plant. The mean diameters of granules were 0.43 mm on day 36, 1.3 mm on day 60, 4.2 mm on day 150 and 6.6 mm on day 230. More details on granular sludge properties and pollutant removal performance in SBR1 have been presented in our previous studies [31]. Images for development of granules with time from SBR1 are shown in Figure 1.

The settling velocity of the granules ranged from 37.4 to 89.7 m/h (activated sludge control group ranged from 4 to 51 m/h). In addition, the average roundness of granules was 1.32 and granular sludge concentration ranged from 8.1 to 10.7 g/L [31]. The results demonstrate that granular sludge had excellent solid–liquid separation due to density, large size, compactness and roundness.

#### 3.1.2. Granules Structure Analysis by CLSM

**Figure 2:**
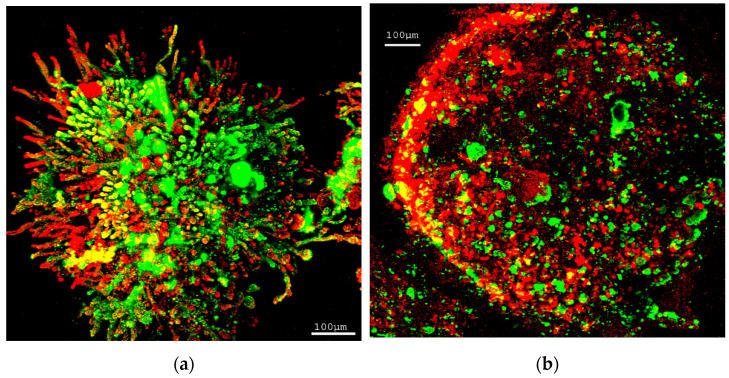
Distribution of bacteria (red) and EPS (green) in small aggregate and granule (diameter < 1 cm). (**a**) A small aggregate; (**b**) a small granule. Scale bar = 100 μm.

Staining with fluorescent labelled lectin for EPS glycoconjugates and with Syto 60 for bacteria was carried out to analyze the distribution of these two components in granules. Figure 2 shows CLSM images of distribution of bacteria (red) and EPS (green) in granules. In a small aggregate during granulation, a number of EPS and filamentous bacteria were found on the surface. In a small granule, alternative distribution of bacteria (red) and EPS (green) was observed on the surface.

**Figure 3:**
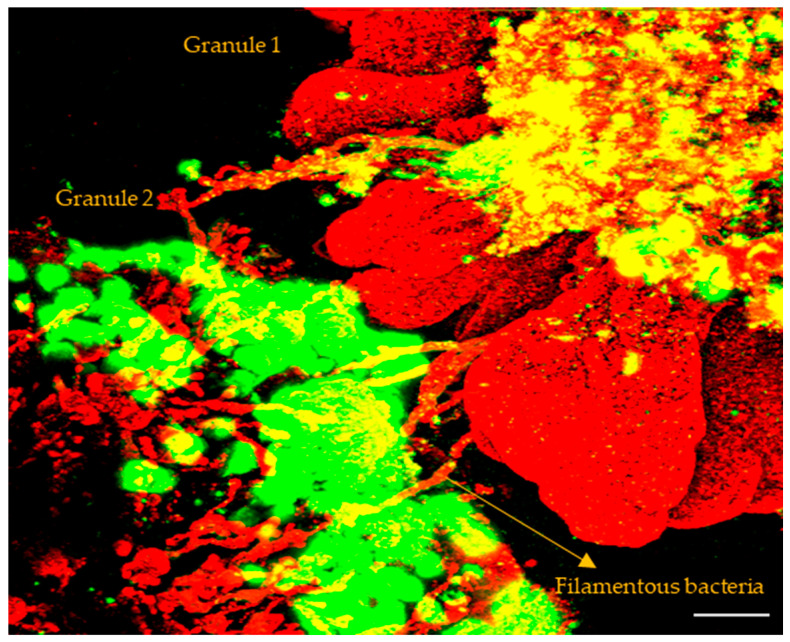
Two tiny bio-particles cohered by bridges of filaments and adhesion of EPS. Bacteria (red) and EPS (green). Scale bar = 50 μm.

By observation of the samples from SBR1, we found some tiny bio-particles and clusters that cohered with each other. Figure 3 shows two tiny bio-particles cohered with filamentous bacteria (which could be bridges) and EPS (which could be adhesive). The left one contains predominantly EPS on the surface, the right one contains lobes of bacteria on the surface. This biological adhesion could promote tiny particles and clusters to aggregate and granulate.

**Figure 4:**
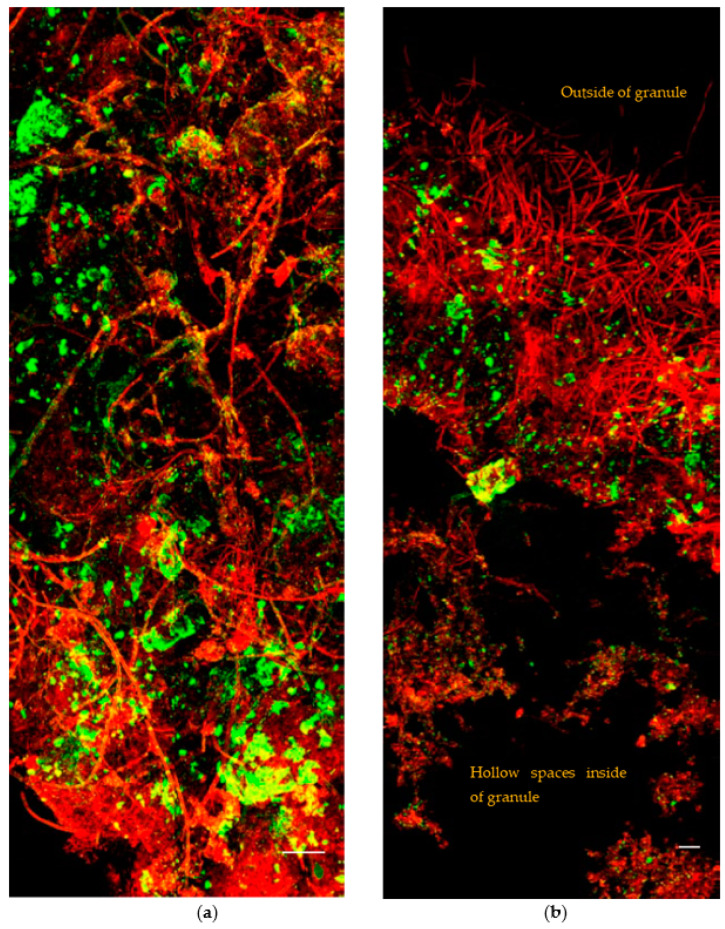
Distribution of bacteria (red) and EPS (green) in large granules (diameter > 6 mm). (**a**) Surface of a large granule; (**b**) partial cross-section of a large granule. Scale bar = 50 μm.

Images of distribution of bacteria (red) and EPS (green) on surface and interior of large granules are shown in Figure 4. We found there were a number of hollow spaces in the interior of large granule, particularly in the core of granules. Filamentous bacteria were distributed on the cross-section of the particles. Nevertheless, the majority of filamentous bacteria were accumulated in the surface layer. These filamentous bacteria seem like a number of networks acting as skeletons to support the structure of granules.

#### 3.1.3. The Broken Granules Structure

With a running time of 240 days, we found that granules were growing and becoming larger and larger in SBR1. The diameter of the largest granule reached 9.6 mm. A question might be proposed: how could these granules with such a large size develop and keep a stable structure? We know aerobic granulation was biological self-aggregation. 

**Figure 5:**
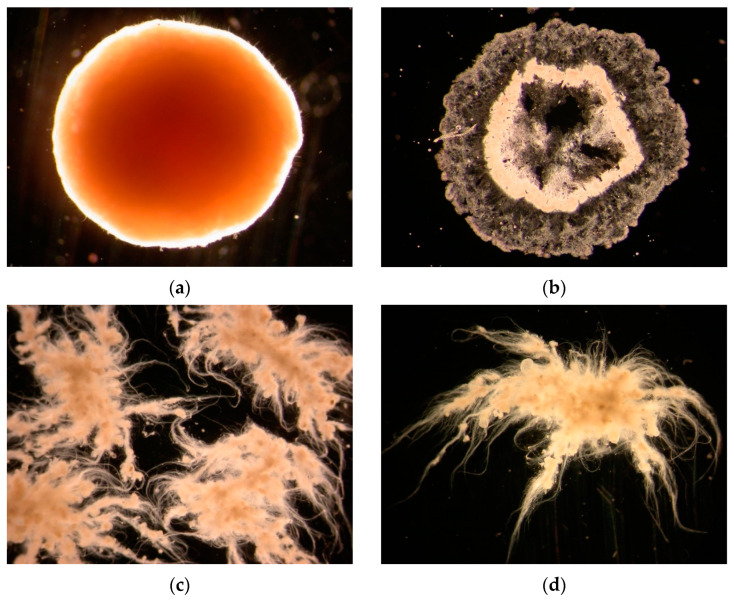
Large granules and broken granules. (**a**) A large granule from SBR1; (**b**) the cross section of a large granule cut by freezing microtome; (**c**) a broken granule torn by a tweezer; (**d**) a piece of torn cluster from a granule. Scale bar = 1 mm.

Figure 5 shows images of a large granule with a diameter of 6.2 mm (a), the cross-section of a large granule cut by freezing microtome (b), a broken granule torn by a tweezer (c) and a piece of torn cluster from a granule (d).

Based on images taken by microscopy, the granules displayed a smooth and round surface structure even though we caught sight of something like fine filaments on the surface. The cross-section of the granule displays three distinguished layers, which include a hollow core, grey middle layer with inert matters and surface layer with a depth of approximately 500–1000 μm. These characteristics coincide with the images observed by CLSM.

Through the microscopy, we observed and used a tweezer to tear granules. Operators could feel the strength of the granules’ structure. The broken granules released something like fine sand, which were inorganic compounds (proven in previous studies) [34,35,36]. We speculate these inorganic matters were produced due to substrates’ transfer resistance [37]. It is more important to find that the anti-broken strength of granules structure was forced by interlaced filamentous bacteria. As shown in Figure 5c,d, the aerobic granular sludge is not completely broken due to tearing, but is divided into several clusters. It can be found that filamentous bacteria cross-grow and intertwine, forming a network structure, and act as skeletons; filling in surface of granules supported the structure of clusters. Therefore, it can be speculated that filamentous bacteria play an important role in the stability of large granular sludge. To a certain extent, these characteristics are similar to the images obtained by CLSM. 

### 3.2. Granules from SBR2 (Domestic Wastewater)

#### 3.2.1. Morphology

**Figure 6:**
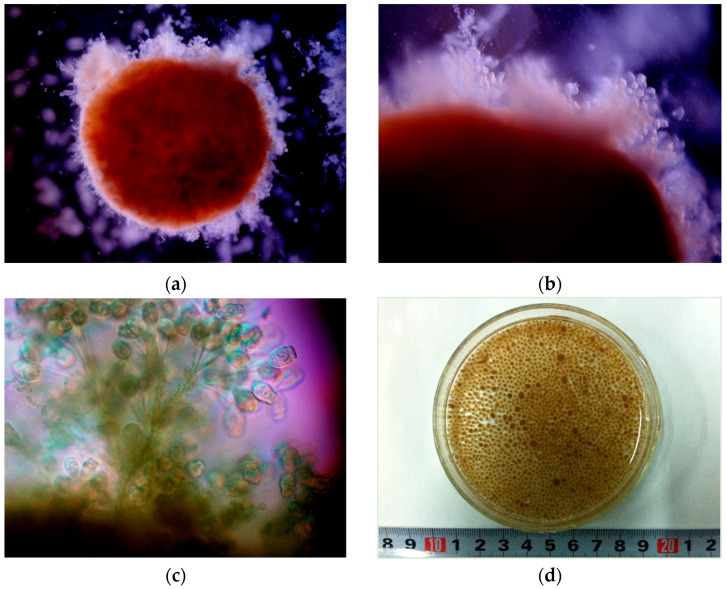
Granules with stalked ciliates (*Epistylis* sp.) in the surface from SBR2 (domestic wastewater). (**a**) A granule from SBR2; (**b**) the surface of a granule; (**c**) *Epistylis* sp. in the surface of a granule; (**d**) granules with stalked ciliates on day 120. Scale bar = 100 μm.

The microscopic examination shown in Figure 6 presents the morphology of mature granules from SBR2. The inoculum was floc sludge from a local municipal wastewater treatment plant. The raw water was real domestic wastewater. The mean diameters of granules were 0.32 mm on day 30, 0.94 mm on day 70 and 2.47 mm on day 120 (more details on granular sludge properties and pollutant removal performance in SBR2 have been presented in our previous studies [12]). Figure 6 displays images of a granule with a diameter of 4.31 mm (a), surface of a granule (b), stalked ciliates (*Epistylis* sp.) in the surface of a granule (c), granules on with stalked ciliates on day 120 (d).

Granule-dwelling Epistylis were not detected earlier than day 30. From images, we can observe the bulky growth of tufted Epistylis rooted in the surface around the granules. Due to the flower-like structure of the surface, the velocity of granule-dwelling Epistylis is lower than that of naked granules.

Stalked ciliates are a type of protozoa which can be branched or unbranched. Stalked ciliates are inverted bell-shaped bodies mounted on a stalk which is attached to a substratum. Epistylis is one genera of stalked ciliates seen commonly in activated sludge for wastewater treatment. Stalked ciliates are usually an indication of a stable activated sludge operation. The species of stalked ciliates found can be used to indicate solids retention time (SRT). The colonial forms of stalked ciliates usually occur at higher SRTs. We speculate that Epistylis occurred in SBR2 is due to real domestic wastewater was used and operated a high SRTs. Stalked ciliates usually anchor themselves to a stable substratum and create a vortex by swirling the water around to swallow single-celled bacteria. This is helpful to promote aerobic sludge granulation and improve effluent quality, because of the removal of wandering and tine-suspended solids.

#### 3.2.2. Granules Structure Analysis by SEM

**Figure 7:**
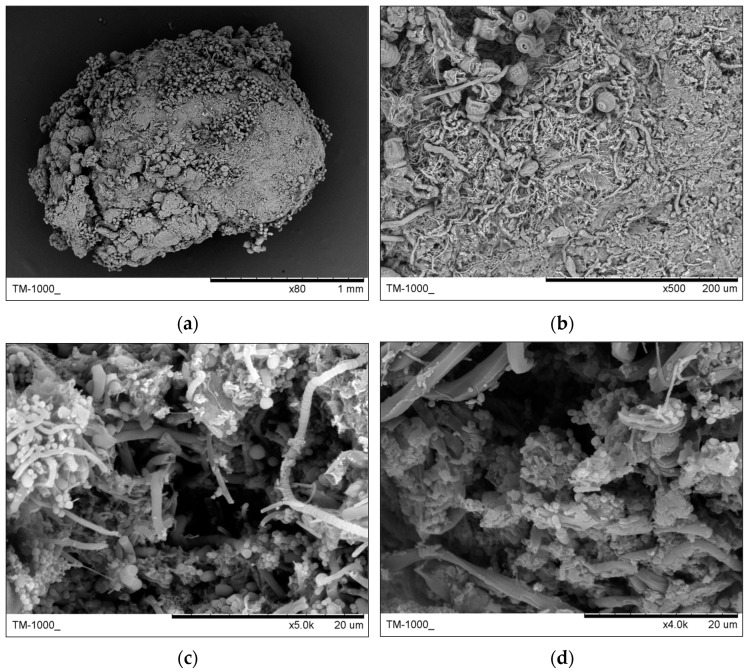
Structure of the granule with stalked ciliates (*Epistylis* sp.) by SEM. (**a**) A granule from SBR2; (**b**) surface layer of the granule; (**c**) middle layer of the granule; (**d**) core layer of the granule.

Images of granules with stalked ciliates taken by SEM for analysis of structure are shown in Figure 7, including a granule with a diameter of 2.16 mm (a), surface layer of the granule colonized stalked ciliates and filamentous bacteria (b), middle layer of the dissected granule (c) and core layer of the dissected granule with hollows (d).

Stalked ciliates anchored themselves to granules by stalks roots, while filamentous bacteria dwelled in granules by interlaced filaments. As granules from SBR1, filamentous bacteria from SBR2 also accumulated dominantly in the surface layer. With an increase in depth into the core of granules, the number of filamentous bacteria decreased.

**Figure 8:**
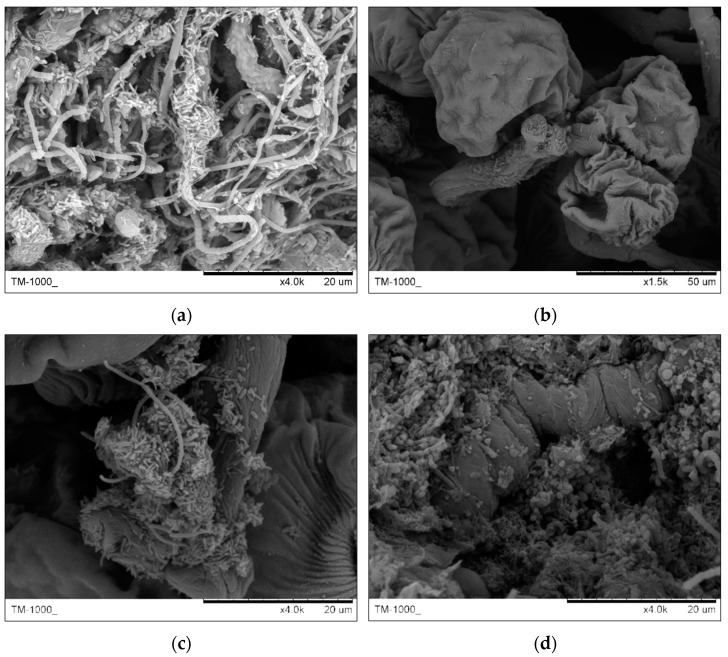
Stalks and filaments acting as skeletons of structure in granules. (**a**) Interlaced structure of stalks and filaments in the surface; (**b**) bell bodies of Epistylis supported by stalks; (**c**) stalk colonized by various bacteria (rods, cocci and filaments); (**d**) dead stalk buried inside of granule.

Images for analysis of stalks and filaments acting as skeletons of the structure in granules are shown in Figure 8, including dense and interlaced structure of stalks and filaments in the surface (a), bell bodies of Epistylis supported by stalks (b), stalk colonized by rods, cocci and filaments (c) and a dead stalk buried inside of granule (d).

This is different to the granules from SBR1 in that the surface layer of these granules from SBR2 were colonized by both stalked ciliates and filamentous bacteria. Both these microorganisms jointly acted as skeletons of granules, particularly supported the stability of structure for large granules.

According to images in Figure 8a,b, the surface of stalked ciliates was colonized by various bacteria, such as rods, cocci and filaments, and the surface of filamentous bacteria was colonized by rods and cocci. Some dead stalks were observed as being buried inside of the granule. We speculate these buried stalks could act as skeletons with the development of granules.

## 4. Discussion

AGS is a promising technology for wastewater treatment due to the transformation of floc sludge to granules. In this study, two types of granules were developed successfully in two SBRs to treat synthetic wastewater (SBR1) and domestic wastewater (SBR2) respectively. A difference between the two types of granules is that no stalked ciliates had been observed in granules from the synthetic wastewater treatment reactor. Domestic wastewater containing particulate organic matter is arguably responsible for the higher recruitment of stalked ciliates from the beginning of the treatment. The hydrolysis of this organic matter leads to the generation of storage polymers [38,39] which further selects for stalked ciliates while restraining filamentous bacteria growth [40,41]. On the other hand, the synthetic wastewater used in this study contained only labile substrates promoting the proliferation of filamentous bacteria [40,42]. Furthermore, the high granular sludge concentration in SBR1 created a lower organic load and higher force of friction. 

AGS can be considered as a special case of biofilm growth without carrier material [43]. The structure of the granules creates dissolved oxygen and substrate concentration gradients along the radial direction, leading to stratification in layers of different types of microorganisms and metabolisms [44]. The large size of the growing granules might threaten the structure stability. 

According to analysis of large granules by CLSM and SEM, filamentous bacteria and stalked ciliates played an important role in structure stability. Diameter of granules from SBR1 and SBR2 reached 9.6 mm and 5.2 mm, respectively. 

Filamentous bacteria are bacteria that produce long mesh-like filaments by growing end-to-end. These bacteria can be seen as long strands under a microscope. They have long thread-like structures such as clumps and chains. They provide a backbone for other bacteria to attach to the surface. They may range in size from 0.8 to 5 µm in width and from 5 to 500 µm in length. In the floc-activated sludge system for wastewater treatment, the presence of some filamentous bacteria is commonly beneficial in floc formation to biomass. Filamentous bacteria act as a base of the structure on which other bacteria can attach and form flocs to aid flocculation and settling of sludge. The filaments could connect to each other and form a mesh for floc formation. Nevertheless, an excessive amount of the filamentous bacteria in wastewater might cause problems of sludge bulking to effect on the separation of sludge and treated water, and may even have impact on wastewater treatment efficiency.

The structure of filamentous bacteria in AGS has a difference somewhat with that in floc-activated sludge. In the initial phase of aerobic sludge granulation, filamentous bacteria attach and bridge the bacteria, promoting the formation of clusters or tiny bio-particles. In large granules, even though hollows occur in the granule’s core, filamentous bacteria as skeletons are accumulated in the surface of the granule to form a space grid structure like a spherical shell to support the stability of granules. These conclusions were proved by the structure analysis of granules from SBR1 and SBR2.

Stalked ciliates of the genus Epistylis belongs to the Phylum Ciliophora [45]. They are about 200–250 µm long and colonies can reach up to 2 mm long. Because stalked ciliates attach to pieces of floc, they usually imply that the biomass (bacteria) is forming well-structured floc that is essential to settling and good effluent quality. The stalk is stuck to the floc so the organism is rooted in the floc. The ciliate stalks serve also as frames for granule development, since bacteria used them as a substratum to grow.

In this study, remnants of stalks were found in the outer parts of the core zone. Depending on the size of the granules, the inner part of the core zone may contain only dead cell debris and hollows. Dead-stalked ciliates leave stalks to serve as fixed support in the granules. These conclusions were proved by structure analysis of granules from SBR2.

**Figure 9:**
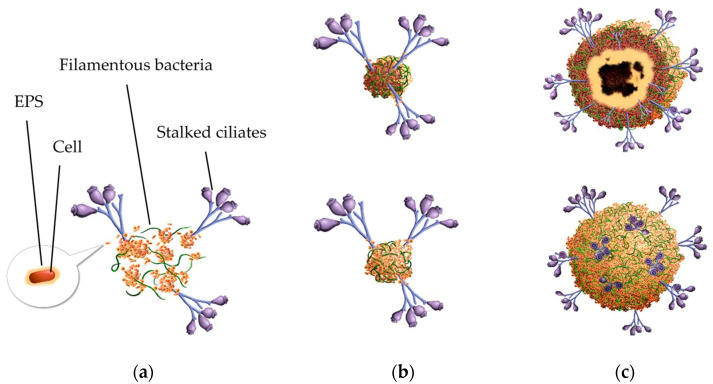
A hypothesis for the roles of filamentous bacteria and stalked ciliates in granule structure. (**a**) Floc sludge; (**b**) a small granule and its cross section; (**c**) a large granule and its cross section.

Based on the analysis of granules, a structure hypothesis is proposed in Figure 9.

## 5. Conclusions

In summary, EPS as a glue, filamentous bacteria and stalked ciliates as skeletons made great contributions to microbial self-aggregation. Filamentous bacteria and stalked ciliates could attach and bridge bacteria to aggregate in initial phase of sludge granulation. In large granules, the space grid structure of filamentous bacteria, or anchoring of stalked ciliates in the surface layer of granules and fixed supports of stalks’ remnants in the interior of granules enhance a stable structure.

## Data Availability

The data presented in this study are available in the article.
